# Identification of a Novel (-)-5-Epieremophilene Synthase from *Salvia miltiorrhiza* via Transcriptome Mining

**DOI:** 10.3389/fpls.2017.00627

**Published:** 2017-04-25

**Authors:** Xin Fang, Chen-Yi Li, Yue Yang, Meng-Ying Cui, Xiao-Ya Chen, Lei Yang

**Affiliations:** ^1^Shanghai Key Laboratory of Plant Functional Genomics and Resources, Shanghai Chenshan Botanical Garden, Shanghai Chenshan Plant Science Research Center, Chinese Academy of SciencesShanghai, China; ^2^State Key Laboratory of Plant Molecular Genetics, CAS Center for Excellence in Molecular Plant Sciences, Shanghai Institute of Plant Physiology and Ecology, Chinese Academy of SciencesShanghai, China

**Keywords:** (-)-5-epieremophilene, natural products, *Salvia miltiorrhiza*, sesquiterpenes, terpene synthases

## Abstract

*Salvia miltiorrhiza*, a medicinal plant in China, has been used for thousands of years to treat coronary heart diseases. Although biosynthesis of tanshinones, a group of diterpenoids in *S. miltiorrhiza*, has been extensively investigated, to date we know little about the formation of monoterpenes and sesquiterpenes in this medicinal plant. Here, we report the characterization of three sesquiterpene synthases, named SmSTPS1, SmSTPS2, and SmSTPS3, which catalyzed the formation of a new compound, (-)-5-epieremophilene. Additionally, the (-)-5-epieremophilene biosynthesis activity of SmSTPS1 was confirmed by transient expression in *Nicotiana benthamiana.* Despite the similar enzyme activities of SmSTPS1, SmSTPS2, and SmSTPS3, the three (-)-5-epieremophilene synthase genes displayed different spatial expression patterns and responded differently to hormone treatments, implicating their specific roles in plant-environment interactions. Our results provide valuable data to understanding the biosynthesis and composition of terpenes in plant.

## Introduction

*Salvia miltiorrhiza*, a perennial herb in the family of Lamiaceae (Menthaceae), is one of the most commonly used medicinal plants in China. Its dry root, known as Danshen in traditional Chinese medicine (TCM), is used in the treatment of coronary heart diseases, particularly angina pectoris and myocardial infarction (Wang et al., 2007). Danshen has been reported to contain two types of active ingredients: the diterpenoid tanshinones and phenolic salvianolic acids (Zhou et al., 2005; Yang et al., 2016).

Lamiaceae is the sixth largest angiosperm family which comprises about 240 genera and more than 7000 species widely distributed in the world (Li et al., 2016). The chemistry of the Lamiaceae plants is dominated by the great diversity and considerable amounts of terpenoids, especially monoterpenes and sesquiterpenes. Within the family, *Salvia* is the largest genus, and plants of this genus are used for medicinal purposes not only in China but also in many other areas. For example, *S. officinalis* is one of the most important medicinal and aromatic plants in Europe, which has antioxidant, antimicrobial, astringent, antihidrotic and specific sensorial properties (Cuvelier et al., 1994; Ghorbanpour et al., 2016; Guneser et al., 2016), and produces rich amounts of monoterpenes and sesquiterpenes (Bouajaj et al., 2013). Root of *S. miltiorrhiza* accumulates a specific group of diterpenoids, known as tanshinones, which are the major active constituents against cerebral infarction (Lin and Hsieh, 2010) and inflammation (Chen and Xu, 2014). In recent years, extensive investigations of biosynthesis of tanshinones have resulted in important progresses. (Gao et al., 2009; Guo et al., 2013, 2016; Cui et al., 2015; Ma et al., 2016). Based on a review of chemical constituents of *Salvia* plants, 46 sesquiterpenoids have been identified from 15 *Salvia* species (Wu et al., 2012). However, until now sesquiterpenes have not been reported from *S. miltiorrhiza*, though more than 40 diterpenoids have been identified.

Plants produce a rich array of terpenoids with diverse structures, which play important roles in both basic biological processes and interactions with environmental factors. Terpene synthases (TPSs), including mono-, sesqui-, and di-terpene synthases, function as key enzymes in biosynthesis of terpenoids, and *TPS* genes often have different expression patterns in different plant tissues. Most of TPSs produce one single dominant product restricted in limited genera or even a single species. For example, (+)-Valencene is a high added-value commercial flavoring responsible for the overall flavor of citrus fruit oils and the corresponding enzyme has been characterized in the genus *Citrus* (Sharon-Asa et al., 2003). Thus, functional characterizations of TPS gene in more species will allow us access to unusual secondary metabolisms previously not encountered. Furthermore, plant secondary metabolisms could be biosynthesized in a tight regulated fashion (Yu et al., 2015) in which the intermediates may be transformed immediately and thus could not be found by traditional phytochemistry way. Genome and transcriptome mining are alternative methodologies developed recently to reveal these inaccessible natural products, such as thalianol (Fazio et al., 2004) and its derives (Castillo et al., 2013) from *Arabidopsis thaliana*. Our previous analysis of *S. miltiorrhiza* transcriptomes has annotated 24 unigenes as TPSs (Yang et al., 2013), most of them have not been functionally characterized. Here, we report three of them, which encode the sesquiterpene synthase for a single product, (-)-5-epieremophilene. To the best of our knowledge, this is the first time (-)-5-epieremophilene and its synthase being reported.

## Materials and Methods

### Plant Materials and Chemicals

Seeds of *S. miltiorrhiza* were collected from Anhui Province and the plants were grown in nursery of Shanghai Chenshan Botanical Garden. For experimental analysis, the plants were grown in greenhouse at 25°C under a light intensity of 150 μmol of photons⋅m^-2^⋅s^-1^ with 14-h-light/10-h-dark cycle. Chemicals were purchased from Sigma-Aldrich, TCI and Fluka.

### Phytohormone Treatment

For hormone treatments, plants of 3-month-old, when biosynthesis of secondary metabolites was active and the transcription levels of the corresponding enzymes were high, were used. The plants were sprayed with the following solutions: 5 mM salicylic acid (SA), 100 μM ethylene (ET), 100 μM abscisic acid (ABA), 50 μM methyl jasmonate (MeJA), and 50 μM gibberellin A_3_ (GA_3_), in addition of a dimethyl sulfoxide (DMSO) solution (5%) as control. Leaf were then collected for isolation of total RNAs 2 h post-spray. Each experiment was performed with at least three biological replicates.

### Plant Terpenoids Extraction

The following materials from the 6-month-old plants in bloom were harvested for extraction of chemicals: primary root (6 cm length from the apical); stem (the second to the fourth internodes from the top); the third pair of true leaves; and the inflorescence. The 6-month-old plants contained a high level of terpenoids and suitable for extraction.

Fresh materials (0.5 g) were ground in liquid nitrogen, followed by adding 2.5 mL hexane to extract the terpenoids in a shaker at 30°C for 1 h. A short column of aluminum oxide overlaid with anhydrous MgSO_4_ was used to purify the extracts, and the obtained hexane phase was analyzed by Gas chromatography–mass spectrometry (GC-MS) by the total ion chromatogram mode.

### cDNA Isolation and Analysis

Total RNAs from the plant tissues were extracted using CTAB solution (2% CTAB, 2% PVP, 100 mM Tris-HCl, 25 mM EDTA, 2M NaCl, 2% β-mercaptoethanol) and treated with DNase I (Takara) according to the manufacturer’s protocol. Total RNA of 1 μg was reverse-transcribed using the ReverTra Ace qPCR RT Kit (TOYOBO, FSQ-101). Rapid amplification of cDNA end (RACE) was performed by the 5′- and the 3′-Full RACE Core Set Ver.2.0 (Takara) according to manufacturer’s instructions. PCR products were cloned into the pMD18-T Vector (Takara) for sequencing. Full-length cDNAs were obtained by assembling fragments obtained by RACE in combination with annotated unigene sequences.

For gene expression analysis, real-time PCR was performed with gene-specific primer pairs on the reverse transcripts derived from total RNAs, using the SYBR^®^ Premix Ex Taq^TM^ II (Perfect Real Time) kit (Takara) on a Mastercycler system (Eppendorf, Germany). *ACTIN* (HM231319) was used as internal reference (Yang et al., 2010). The relative expression value was calculated via the 2^-ΔΔCt^ method. All PCR primers used in this investigation are listed in **Supplementary Table S1**.

Terpene synthase sequences from other plant species were obtained through searching the GenBank database at NCBI. Alignment of protein sequences was performed with CLUSTAL W program of MEGA 6 software with default parameters. Phylogenetic tree was built with the neighbor-joining method with MEGA 6 program. Chloroplast signal peptide prediction was performed by SignalP^1^ software.

### Prokaryotic Expression and Protein Purification

The open reading frames of cDNAs were amplified with fast-*Pfu* DNA polymerase, the PCR products were then digested by *EcoR*I and *Hind*III, followed by ligation into *pET-32a* expression vector. After confirmation by DNA sequencing, the plasmids were transferred into *Escherichia coli* Rosetta (DE3) for protein production. Recombinant proteins were purified with Ni-NTA resin (Thermo) according to manufacturer’s manual. Protein concentrations were determined with the Bradford method using BSA as standard.

### Enzyme Assay

The TPS activity was assayed as described (Ruan et al., 2016). The reaction was conducted in a volume of 500 μL buffer containing 20 μg purified recombinant protein, 25 mM hydroxyethyl piperazineethanesulfonic acid (HEPES, pH 7.0), 5 mM MgCl_2_, 5 mM dithiothreitol (DTT) and 40 μM FPP, and incubated at 30°C for 1 h unless otherwise indicated. The reaction mixture was extracted with 800 μL hexane and subjected to analysis by GC-MS. To determine the quantities of the products, nonyl acetate (10 ng/μL) was added to the hexane as internal standard.

Kinetic parameters of the enzymes were determined as described (Ruan et al., 2016). Briefly, the purified recombinant enzyme (5 μg) was added to each assay mixture containing FPP range from 2.3 to 184.6 μM. The reaction was allowed to proceed for 5 min at 30°C, stopped by the addition of 0.5 M EDTA (pH 8.0) and the reaction products were extracted with 800 μL hexane containing 10 ng/μL nonyl acetate as internal standard, followed by GC-MS analysis. Kinetic parameters were obtained with GraphPad Prism 5 software fitting the Michaelis–Menten curve.

### (-)-5-Epieremophilene Purification

To obtain enough amount of enzymatic product, a 500 mL reaction buffer contained 25 mM Hepes (pH 7.0), 5 mM MgCl_2_, 5 mM DTT, 80 μM FPP and 20 mg purified protein was used. The reaction mixture was overlaid with 20 mL HPLC-grade pentane and incubated at 30°C overnight. The mixture was extracted with 3 × 100 mL pentane, the combined extracts was evaporated under reduced pressure to afford 4.5 mg sesquiterpene product.

### Chemical Analyses

^1^H, ^13^C and 2D nuclear magnetic resonance (NMR) spectra were recorded on a Bruker AVANCEIII^TM^ 500 spectrometer. Chemical shifts were reported using tetramethylsilane as the internal standard. The optical rotation was determined by using a autopol I spectrometer (rudolph research analytical).

### Transient Expression in *Nicotiana benthamiana*

*Nicotiana benthamiana* plants were grown in greenhouse, and 6-week old plants were used for the transient expression. The full-length coding region of *SmSTPS1* was amplified by PCR with the primers listed in **Supplementary Table S1**, the PCR products were firstly cloned into the entry vector pDonr207 and then into plasmid pEAQ-*HT*-DEST1 using the Gateway BP and LR Clonase II enzyme mix (Thermo Fisher), respectively. The expression vector pEAQ-SmSTPS1 was introduced into *Agrobacterium* (strain GV3101). For transient transformation, *Agrobacterium* cells harboring pEAQ-SmSTPS1 was infiltrated into the abaxial side of healthy leaves of *N. benthamiana* using a syringe. After culture in the greenhouse for 4 days, the infected areas were extracted and the chemicals were detected by GC-MS.

### Gas Chromatography–Mass Spectrometry Analysis

Gas chromatography–mass spectrometry analysis was carried out on Agilent 6890 Series GC System coupled to an Agilent 5973 Network Mass Selective Detector, with the carrier gas helium at 1 mL/min, splitless injection, a agilent HP-5MS column (5% phenyl methyl siloxane, length 30.0 m, diameter: 250.00 μm, film thickness: 0.25 μm) for non-chiral analysis. For volatile terpenes detection of *S. miltiorrhiza*, a temperature program from 60°C (5 min hold) at 5°C/min to 260°C was used; For enzymatical assay, the following temperature program was used: initial temperature of 60°C (5 min hold), increase to 250°C by 10°C/min, and ramp to 300°C by 50°C/min (5 min hold). Compounds were identified by comparison with NIST data base (National Institute of Standards and Technology) library and retention indices (Garms et al., 2010).

## Results

### Volatile Terpenes of *S. miltiorrhiza* Determined by GC-MS

**FIGURE 1:**
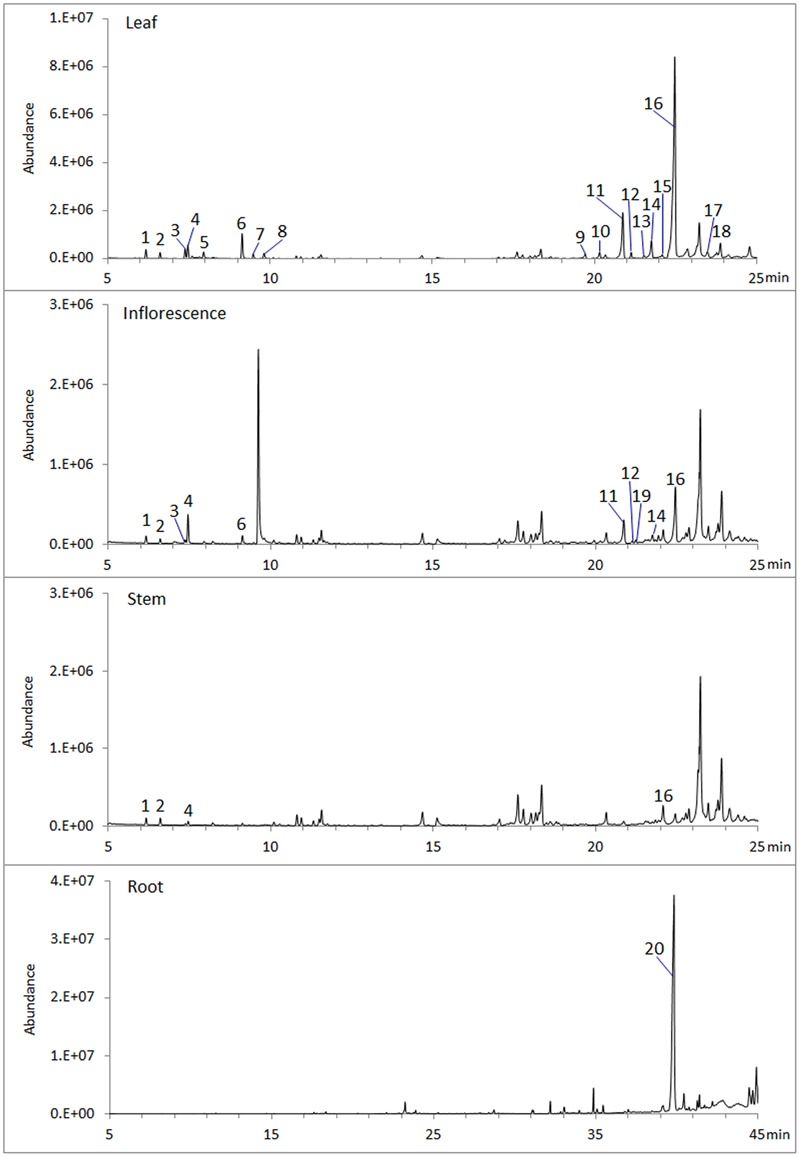
**Volatile terpenes detected from *S. miltiorrhiza* plant.** Samples of root, stem, leaf, and inflorescence, 0.5 g (fresh weight) each, were extracted with 2.5 mL hexane for 1 h at room temperature and analyzed by GC-MS. Peaks are: 1, α-pinene; 2, Camphene; 3, sabinene; 4, β-pinene; 5, β-myrcene; 6, β-thujene; 7, *trans*-β-ocimene; 8, *cis*-β-ocimene; 9, α-copaene; 10, β-elemene; 11, β-caryophyllene; 12, β-copaene; 13, cadina-3.5-diene; 14, α-humulene; 15, *trans*-cadina-1(6),4-diene; 16, germacrene D; 17, β-cadinene; 18, germacrene D-4-ol; 19, germacrene B; 20, ferruginol.

To analyze volatile terpenes in root, stem, leaf and inflorescence of *S. miltiorrhiza*, fresh tissues were extracted with hexane and subjected to GC-MS. In total 20 terpenes were detected, including 8 monoterpenes, 11 sesquiterenes, and 1 diterpenes (**Figure 1**). Of the four organs sampled, leaf contained the most diversified terpenes, with at least 18 compounds (8 monoterpenes, 10 sesquiterpenes) being identified by searching NIST database and retention indices (Garms et al., 2010). By contrast, root had the lowest diversity, as GC-MS identified only 1 diterpene (ferruginol). Notably, ferruginol was not present at a detectable level in the aerial tissues examined.

### Isolation of Terpene Synthase Genes and Phylogenetic Analysis

**FIGURE 2:**
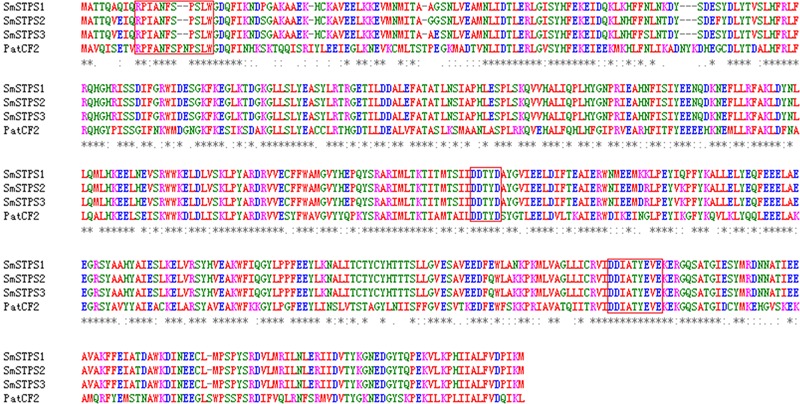
**Alignment of deduced amino acid sequences of SmSTPS1, SmSTPS2, SmSTPS3 with *Pogostemon cablin* germacrene A synthase, PatTpsCF2 (AY508728).** The modified RRx_8_W and DDXXD motifs and the metal binding motif NSE/DTE domain are framed. The sequences were aligned by Clustal W.

We searched *S. miltiorrhiza* transcriptomes (Yang et al., 2013) for TPSs, and amplified cDNAs by 5′- and 3′-RACE. Three cDNAs, namely *SmSTPS1, SmSTPS2*, and *SmSTPS3* (GenBank accession numbers are KY432512, KY432513, and KY432514), were isolated. They all encode proteins of 546 amino acids, which are highly identical to each other with the sequence identity of 97∼99%. Based on *S. miltiorrhiza* genome sequence database (Xu et al., 2016), *SmSTPS1, SmSTPS2*, and *SmSTPS3* are duplicates arranged as a cluster in the genome. Searching of National Center for Biotechnology Information (NCBI) non-redundant protein database showed that the three proteins share a high (62%) sequence identity to germacrene A synthase PatTpsCF2 (AY508728) from *Pogostemon cablin* (Deguerry et al., 2006), another Lamiaceae species which is particularly rich in sesquiterpenes (Swamy and Sinniah, 2015). The three proteins of *S. miltiorrhiza* contain the conserved domain DDxxD involved in the coordination of divalent ions and water molecules, and a modified NSE/DTE domain [ND]D[LIV]x[ST]x_3_E at the *C*-terminal involved in metal cofactor binding (Whittington et al., 2002). In addition, a modified RRx_8_W motif exists at the *N*-terminal (**Figure 2**), which is related to cyclic products formation (Martin et al., 2010; Liu et al., 2014).

**FIGURE 3:**
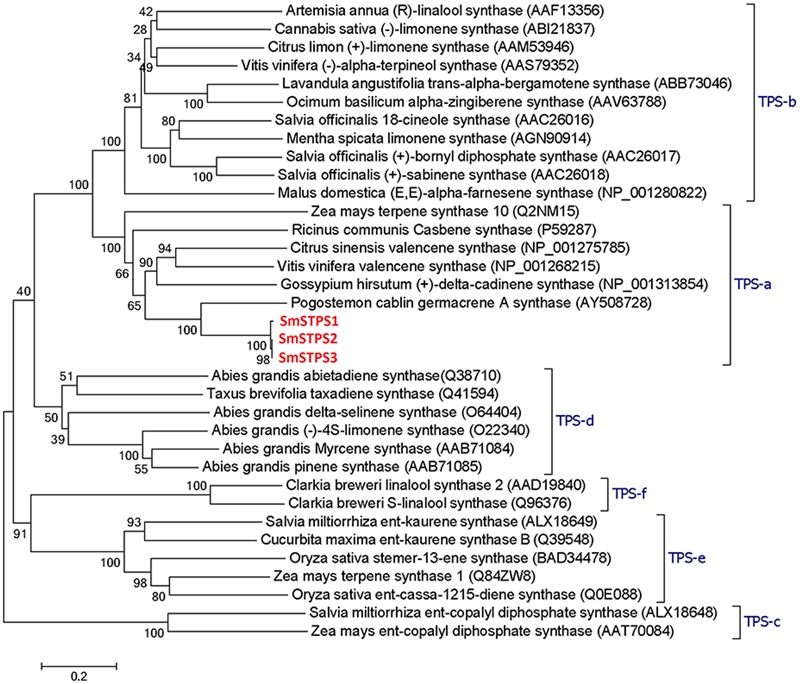
**Phylogenetic analysis of three sesquiterpene synthases from *Salvia miltiorrhiza* with other plant terpene synthases of different clades.** In TPS-a the three *S. miltiorrhiza* (-)-5-epieremophilene synthases share high identities with *Pogostemon cablin* germacrene A synthase. The phylogenetic tree was constructed by Dayhoff’s distances between proteins using the neighbor-joining method. Scale bar indicates 0.2 amino acid substitutions per site. Numbers are the actual bootstrap values of branches. The Protein ID in NCBI protein database are given in parentheses.

Terpene synthases of plants are divided into eight clades or subfamilies designated TPS-a through TPS-h, with a minimum identity of 40% in each clade, and most angiosperm sesquiterpene synthases are clustered into TPS-a subfamily (Bohlmann et al., 1997; Pazouki and Niinemets, 2016). SmSTPS1, SmSTPS2, and SmSTPS3 appear to be typical sesquiterpene synthases (**Figure 3**).

### Enzyme Activities of the Three TPSs

**FIGURE 4:**
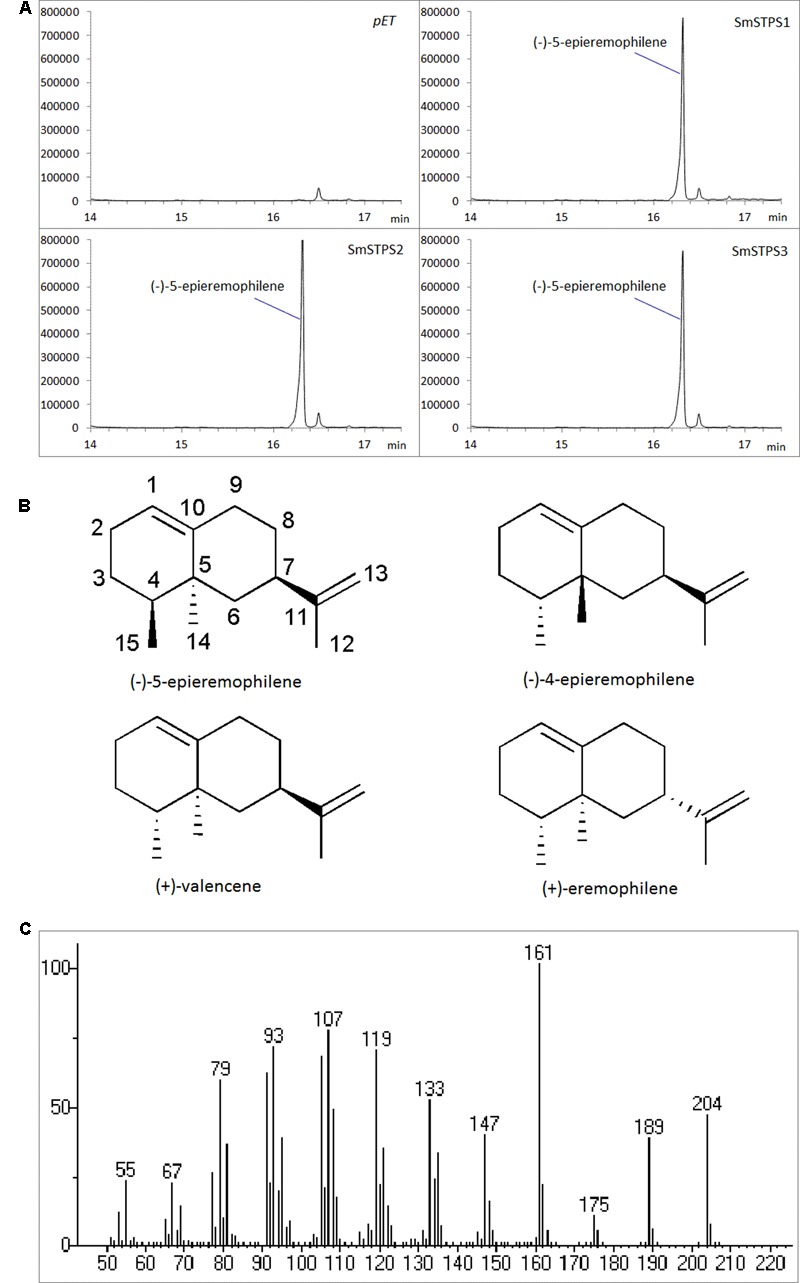
**Identification of the products catalyzed by recombinant SmSTPS1, SmSTPS2, and SmSTPS3 *in vitro*. (A)** Total ion chromatograms from GC-MS analysis of hexane extracts of SmSTPS1, SmSTPS2, and SmSTPS3 after incubation with FPP as substrate. The protein tag produced by *pET32a* was used as control. **(B)** Chemical structure of the identified compound (-)-5-epieremophilene and structural related compounds. **(C)** Mass spectrum of the identified (-)-5-epieremophilene.

The three putative terpene synthases were then expressed in *E. coli* cells (**Supplementary Figure S1**). The purified fusion proteins were incubated with farnesyl diphosphate (FPP) as the substrate, and Mg^2+^ as a metal cofactor. SmSTPS1, SmSTPS2 and SmSTPS3 all converted FPP into a single and same 204 Da sesquiterpene product, corresponding to the chemical formula C_15_H_24_ (**Figure 4**). Its MS spectrum is closely resemble those of valencene by searching NIST database, however, the retention time of this enzymatic product in GC-MS analysis is different to that of authentic (+)-valencene (**Supplementary Figure S2**), suggesting that it may share the same skeleton with valencene.

Kinetic analysis with FPP in the presence of Mg^2+^ showed that the *K_m_* values of SmSTPS1, SmSTPS2 and SmSTPS3 are 25.35, 24.20, and 10.44 μM, the estimated *k_cat_* are 2.09 s^-1^, 2.07 s^-1^ and 1.53 s^-1^, and the *k_cat_/K_m_* values are 8.27 × 10^4^ s^-1^⋅M^-1^, 8.56 × 10^4^ s^-1^⋅M^-1^, and 1.48 × 10^5^ s^-1^⋅M^-1^, respectively (**Table 1**). Thus, these three synthases have similar catalytic activities, though SmSTPS1 and SmSTPS2 are slightly more efficient. These parameters are in the normal range of plant TPSs.

**Table 1:** Kinetic Parameters of SmSTPS1, SmSTPS2, and SmSTPS3.

	*Km* (μM)	*kcat* (s^-1^)	*kcat*/*Km* (s^-1⋅^M^-1^)
SmSTPS1	25.35 ± 1.88	2.09 ± 0.05	8.27 × 10^4^ ± 4.06 × 10^4^
SmSTPS2	24.20 ± 0.84	2.07 ± 0.13	8.56 × 10^4^± 3.82 × 10^4^
SmSTPS3	10.44 ± 1.07	1.53 ± 0.02	1.48 × 10^5^± 1.32 × 10^4^

*SmSTPS1, SmSTPS2, and SmSTPS3 were expressed in *E. coli* as recombinant proteins, and the enzyme activity was assayed at 30°C for 5 min with FPP as substrate.*

### Structural Analysis of the Enzymatic Product

To elucidate the structure of the enzymatic product of three putative terpene synthases, we collected 4.5 mg this compound through large scale incubation experiment. The ^1^H and ^13^C NMR spectroscopic data of this sesquiterpene product (**Table 2**) were analogous to those of (+)-eremophilene (Schifrin et al., 2015), a stereoisomer of (+)-valencene, but their MS data are different; furthermore, in GC-MS separation of (+)-eremophilene and (+)-valencene mix, peak of (+)-valencene eluted before (+)-eremophilene (Schifrin et al., 2015), whilst in our GC-MS analysis of (+)-valencene and this esequiterpene product mix, (+)-valencene eluted after this compound (**Supplementary Figure S2**). All of these suggest that these compounds are structurally similar. The detailed 2D NMR analysis allowed the construction of the structure of the sesquiterpene (**Supplementary Figures S3–S8**). The ^1^H-^1^H chemical shift correlation spectroscopy (COSY) correlation of an olefinic proton (δ_H_ = 5.36) to H-2, and its heteronuclear multiple bond correlation (HMBC) correlations to C-3, and C-9 indicate the presence of C1-C10 double bound. The other olefinic protons (δ_H_ = 4.74, *J* = 15 Hz) have HMBC cross peaks with C-7 and C-13 methyl group, locating the exo-isopropylidene group at C-7. The HMBC correlations of C-15 methyl group to C-3, C-4, and C-5; And C-14 methyl group to C-4, C-5, C-6, and C-10 attached these two methyls at C-4 and C-5, respectively. Thus, this compound has the same planar structure as valencene and eremophilene, which harbors three chiral centers of C-4, C-5, and C-7, indicating the existence of eight stereoisomers.

**Table 2:** ^1^H (500 MHz) and ^13^C (125 MHz) NMR spectroscopic data for (-)-5-epieremophilene in CDCl_3_.

Position	^13^C [δ in ppm]	^1^H [δ in ppm, m]
1	120.51	5.36 m
2a/b	25.41	2.07 m; 1.97, m
3	27.09	1.47 m, 2 H
4	37.05	1.53 m
5	38.11	–
6α/β	39.76	2.00 m; 1.55, m
7	38.51	2.06 m
8	30.11	1.69 m, 2H
9β/α	28.40	2.41 m; 1.97 m
10	144.08	–
11	150.11	–
12	21.53	1.75 br s, 3H
13	108.33	4.74 d, *J* = 15 Hz, 2H
14	20.23	0.94 s, 3H
15	16.02	0.89 d, *J* = 10 Hz, 3H

The GC-MS and NMR data of this compound are different to those of (+)-valencene, (-)-4-epieremophilene (Zhao et al., 2004) and (+)-eremophilene (Schifrin et al., 2015), and thus their enantiomers (-)-valencene, (+)-4-epieremophilene, and (-)-eremophilene, only leaving (+)-5-eremophilene and (-)-5-eremophilene as two possible candidates. The rotating frame overhauser effect spectroscopy (ROESY) data are in agreement with this assignment. The optical rotation analysis gave a [α]^24^ value of -29.4 (ethanol). Taken together, this compound was unambiguously determined as (-)-5-epieremophilene, which is neither isolated nor synthesized previously. And these three sesquiterpene synthases are *S. miltiorrhiza* (-)-5-epieremophilene synthases.

### Transient Expression of SmSTPS1 in *Nicotiana benthamiana*

**FIGURE 5:**
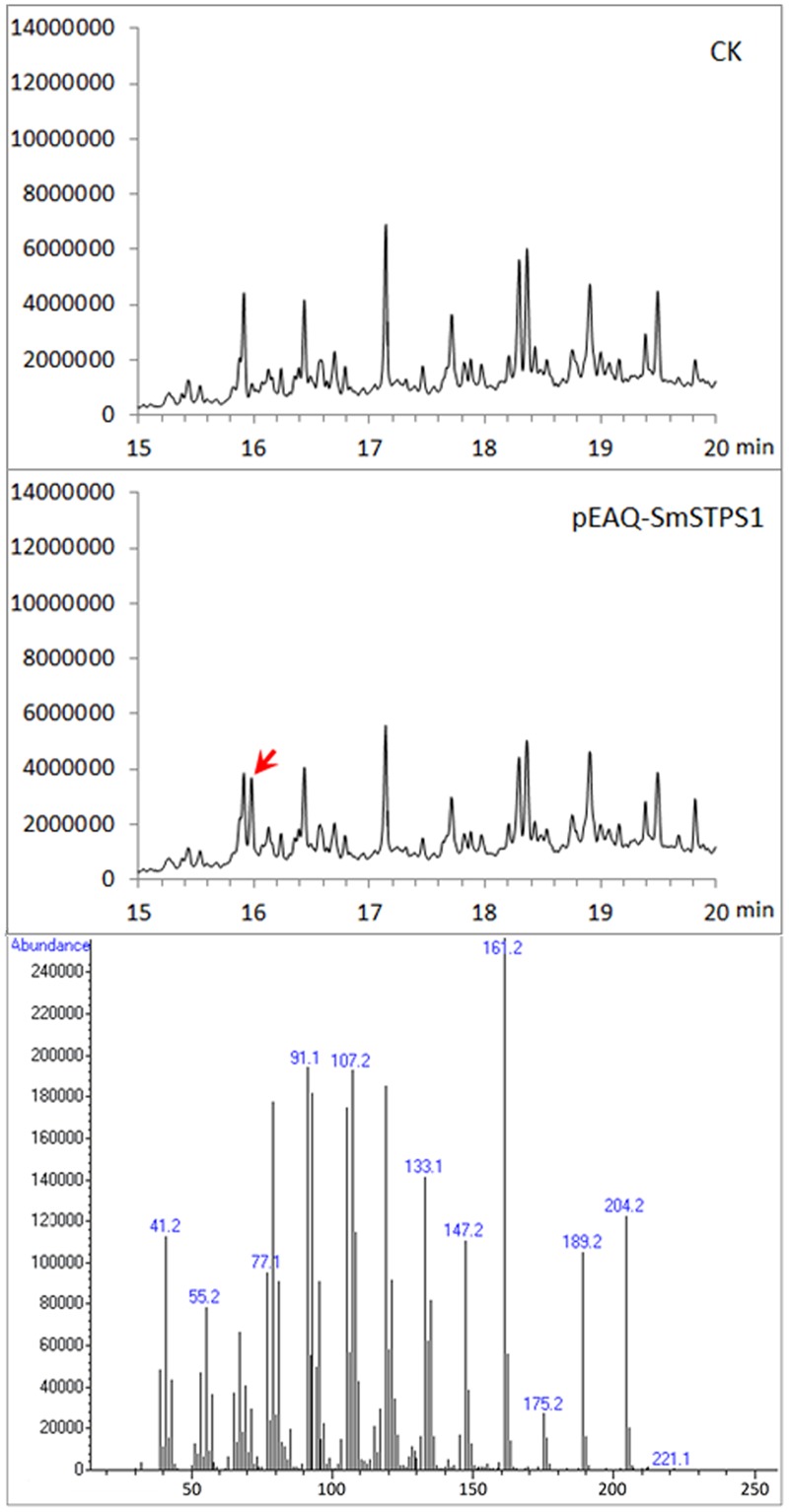
**Production of (-)-5-epieremophilene in *N. benthamiana* leaves transiently expressing *SmSTPS1*.** The leaves were infiltrated with *Agrobacterium* cells harboring pEAQ-SmSTPS1, or the empty pEAQ as control, the infected areas were extracted 4 days post-infiltration, and the extracts were analyzed by GC-MS. Red arrow indicates the peak of (-)-5-epieremophilene, and the MS spectrum of this peak is given.

Because (-)-5-epieremophilene was not detected from the extracts of *S. miltiorrhiza* plants, we adopted a transient expression system to produce recombinant proteins in leaves of *N. benthamiana* (Peyret and Lomonossoff, 2013; Sainsbury and Lomonossoff, 2014), in order to further assay the activity of *S. miltiorrhiza* (-)-5-eiperemophilene synthase *in planta*. After 4 d of the infiltration of *Agrobacterium* harboring pEAQ-SmSTPS1, the leaves were gathered and extracted. We found that, compared to the extracts from control *N. benthamiana* leaves, an extra peak with the retention time of approximately 16 min appeared when SmSTPS1 was expressed, which was determined to be (-)-5-epieremophilene by GC-MS (**Figure 5**).

### Spatial Expression Patterns and Hormone Induction

**FIGURE 6:**
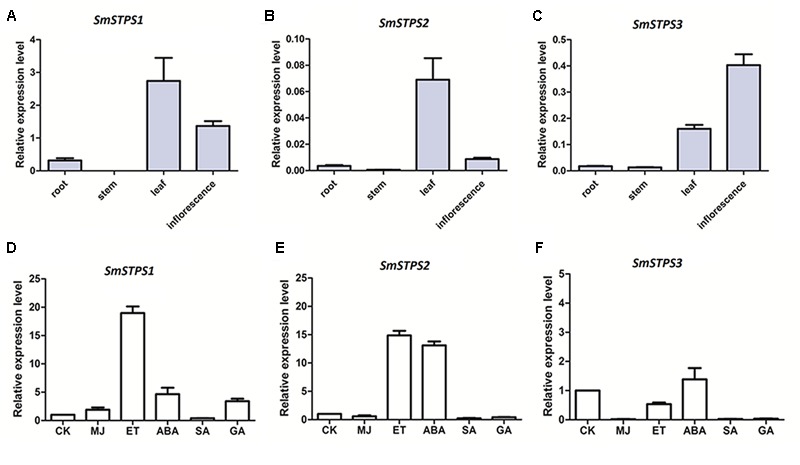
**Relative expressions of *SmSTPS1, SmSTPS2*, and *SmSTPS3* in different organs of *S. miltiorrhiza* and their induction by hormone treatments.** The transcripts were analyzed by quantitative real-time RT-PCR, with *SmACTIN* (HM231319) as internal standard. **(A–C)** Relative expression levels of *SmSTPS1, SmSTPS2*, and *SmSTPS3* in root, stem, leaf, and inflorescence. **(D–F)** Relative expression levels after hormone treatments. Plants were sprayed with 5 mM SA, 100 μM ET, 100 μM ABA, 50 μM MeJA, and 50 μM GA_3_, or a DMSO solution as control, respectively. Leaves were collected 2 h post-treatment for RNA isolation. The expression level of each gene in the control sample (CK) is set to 1, error bars indicate standard deviation (SD) of three replicates.

Relative expression levels *SmSTPS1, SmSTPS2*, and *SmSTPS3* in root and aerial organs were analyzed by quantitative real-time PCR (qRT-PCR). The three (-)-5-epieremophilene synthase genes were mainly expressed in leaf and inflorescence, and their transcript levels were low or undetectable in stem and root. In general, *SmSTPS1* was expressed at a higher level than the other two, and its transcripts were most abundant in leaf. *SmSTPS2* was weakly expressed and exhibited a clear expression only in leaf, whereas *SmSTPS3* was expressed predominantly in inflorescence (**Figures 6A–C**). Thus, the three *STPS* genes were differentially regulated in *S. miltiorrhiza* plants.

Next, we examined responses of the three sesquiterpene synthase genes to phytohormone treatments. Since *SmSTPS1, SmSTPS2*, and *SmSTPS3* were all expressed in *S. miltiorrhiza* leaves, changes in their expression levels in leaves were analyzed after hormone treatments. We found that, although the three sesquiterpene synthases share high similarities in sequences and catalytic activities, they responded differentially to different phytohormones at transcription level. *SmSTPS1* was induced strongly by ethylene (ET) and moderately by MeJA, abscicic acid (ABA) and gibberellin A_3_ (GA_3_) (**Figure 6D**), *SmSTPS2* responded not only to ET but also to ABA (**Figure 6E**), and *SmSTPS3* was up-regulated slightly only by ABA (**Figure 6F**).

## Discussion

To date, more than 700 secondary metabolites have been reported from plants of *Salvia*, most of which (>80%) are terpenes (Wu et al., 2012). In *S. miltiorrhiza* and closely related species distributed in China diterpenoids are dominant in root (Li et al., 2008). Other natural products, such as flavonoids, were also reported from a number of species of the genus (Cuvelier et al., 1994). In this study, we analyzed volatile terpenes in different parts of *S. miltiorrhiza*, and found that root is distinct from aerial organs in accumulating much less volatile terpenes. In Arabidopsis, 20 of the 32 terpene synthase genes annotated were found to be expressed in flowers, which are responsible for the emission of both monoterpenes and sesquiterpenes (Chen et al., 2003). Microarray expression profiling also showed the distinct patterns of *AtTPS* genes in different organs (Ro et al., 2006). The diterpene ferruginol detected in *S. miltiorrhiza* root is an intermediate in the biosynthetic pathway to tanshinones (Guo et al., 2013; Ma et al., 2016), which are mainly synthesized in root (Yang et al., 1996). Our results demonstrate that *S. miltiorrhiza* synthesizes and accumulates distinct terpene compositions in different organs, suggesting a strategy of enhancing adaptation to various environmental factors through using diverse secondary metabolites, consistent with the report of Eucalyptus, which has a highly diverse family of TPS genes differentially regulated to generate unique terpene profiles in different parts of the plant (Kulheim et al., 2015).

Aroma is characteristic of Lamiaceae family, and the volatiles with biological activities are often the major interests of the resources. Terpenes are often the important constituents of plant essential oils and have a variety of biological functions, among which sesquiterpenes form the largest group and are greatly valuable for their aromatic and pharmaceutical properties. *S. miltiorrhiza* is an important medicinal plant in China; differing from the European species *S. officinalis*, it has no particular scent even from the flowers. The three *S. miltiorrhiza* sesquiterpene synthases characterized here, SmSTPS1, SmSTPS2, and SmSTPS3, share a high sequence identity to PatTpsCF2 (AY508728) that catalyzes the biosynthesis of (+)-germacrene A as the dominant product with germacrene D, 4,5-di-*epi*-aristolochene, α-selinene and eremophilene as side products (Deguerry et al., 2006). The SmSTPS1, SmSTPS2, and SmSTPS3 reported here function as (-)-5-epieremophilene synthase *in vitro*, and the SmSTPS1 activity of synthesizing (-)-5-epieremophilene *in planta* was confirmed by transient expression. However, (-)-5-epieremophilene was not detected from *S. miltiorrhiza* in our experiments, neither was it reported from species of *Salvia*. One possibility is that there is a biosynthesis pathway that transforms (-)-5-epieremophilene into other non-volatile product(s), which awaits identification. Importantly, the transcriptome mining approach has enabled us to identify this previously unknown metabolite, suggesting a great potential of this method in further exploring the treasure of plant metabolites.

Sesquiterpenes bearing valencene backbone have been isolated from different plant species and attracted intensive attention due to their broad activity. (+)-Valencene was reported to significantly inhibit the activity of transient receptor potential ankyrin-1 (TRPV1) and calcium release-activated calcium modulator 1 (ORAI1), which are critical in mediating UV-induced skin aging, thus this natural product is suggested to be a potent candidate for the development of therapeutic agents for the prevention and treatment of UV-induced photoaging (Nam et al., 2016). Its oxidative product, (+)-nootkatone, has also received interests in pharmaceutical industry for its anti-platelet aggregation effects (Choi et al., 2014) and anti-proliferative activity toward cancer cells (Gliszczynska et al., 2011), as well as its activity in repelling insects (Zhu et al., 2001). As (-)-5-epieremophilene is identified and can be produced by the three (-)-5-epieremophilene synthases from *S. miltiorrhiza* efficiently, it would be interesting to compare the activities of these structurally related sesquiterpenes.

## Author Contributions

LY and XF designed the study and wrote the manuscript. LY, YY, C-YL, and M-YC performed the experiment. X-YC helped in data analysis and manuscript preparation.

## Conflict of Interest Statement

The authors declare that the research was conducted in the absence of any commercial or financial relationships that could be construed as a potential conflict of interest.

## Footnotes

1 http://www.cbs.dtu.dk/services/SignalP/

## Supplementary Material

FIGURE S1 **Analysis of recombinant proteins. (A–C)** SDS-PAGE of recombinant SmSTPS1, SmSTPS2, SmSTPS3 (1: 0 mM IPTG; 2: 0.2 mM IPTG; 3:0.5 mM IPTG; 4: 1 mM IPTG).

FIGURE S2 **Total ion chromatograms from GC-MS analysis of the enzymatic product and authentic (+)-valencene**.

FIGURE S3 **^1^H NMR spectra of (-)-5-epieremophilene**.

FIGURE S4 **^13^C NMR spectra of (-)-5-epieremophilene**.

FIGURE S5 **HSQC NMR spectra of (-)-5-epieremophilene**.

FIGURE S6 **^1^H-^1^H COZY NMR spectra of (-)-5-epieremophilene**.

FIGURE S7 **Heteronuclear multiple bond correlation HMBC NMR spectra of (-)-5-epieremophilene**.

FIGURE S8 **Rotating frame overhauser effect spectroscopy NMR spectra of (-)-5-epieremophilene**.

TABLE S1 **List of oligonucleotide primers.** All primers used for full-length cDNA cloning and qRT-PCR analyses in this investigation are listed and their sequences are given.
