# Supplementary figures and images for: Identification of a Novel (-)-5-Epieremophilene Synthase from Salvia miltiorrhiza via Transcriptome Mining
Source: Front Plant Sci. 2017 Apr 25;8:627. doi: 10.3389/fpls.2017.00627 (PMC5404360; doi:10.3389/fpls.2017.00627)

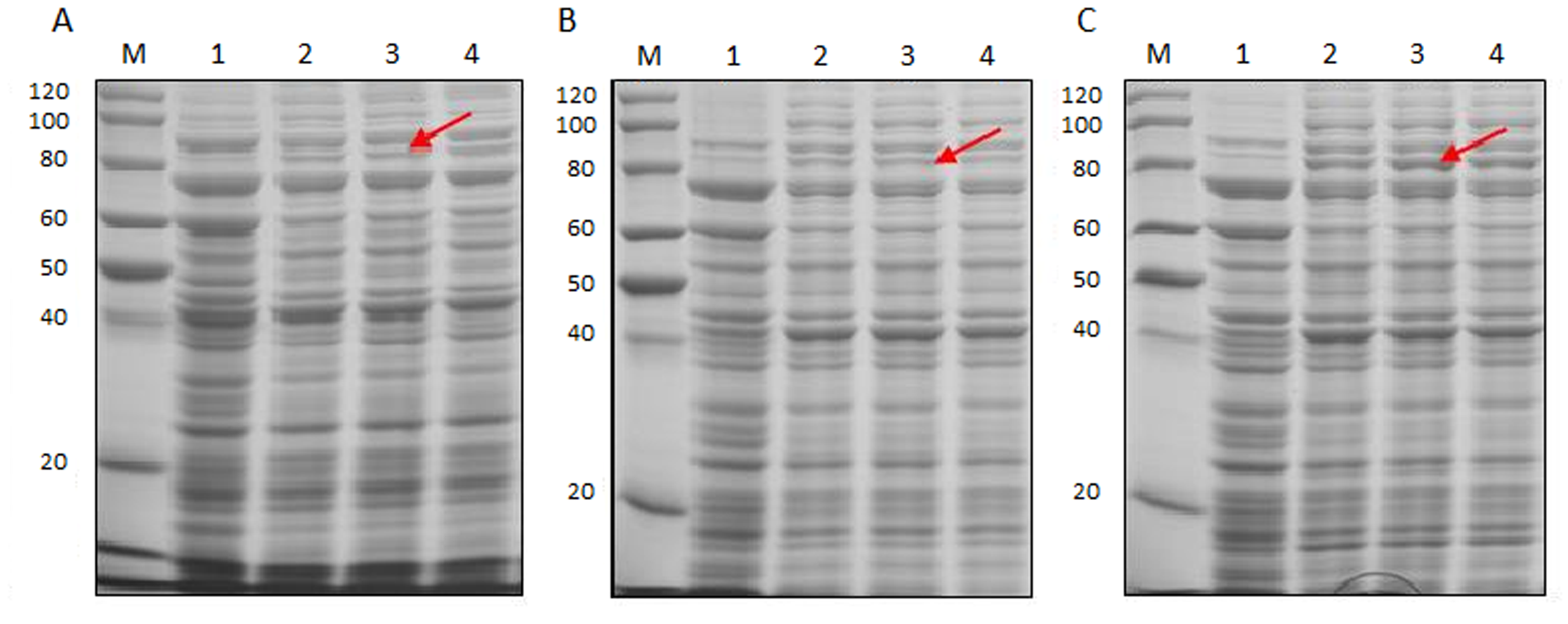

Supplement: FIGURE S1 — Analysis of recombinant proteins. (A–C) SDS-PAGE of recombinant SmSTPS1, SmSTPS2, SmSTPS3 (1: 0 mM IPTG; 2: 0.2 mM IPTG; 3:0.5 mM IPTG; 4: 1 mM IPTG). [file Image_1.TIF]

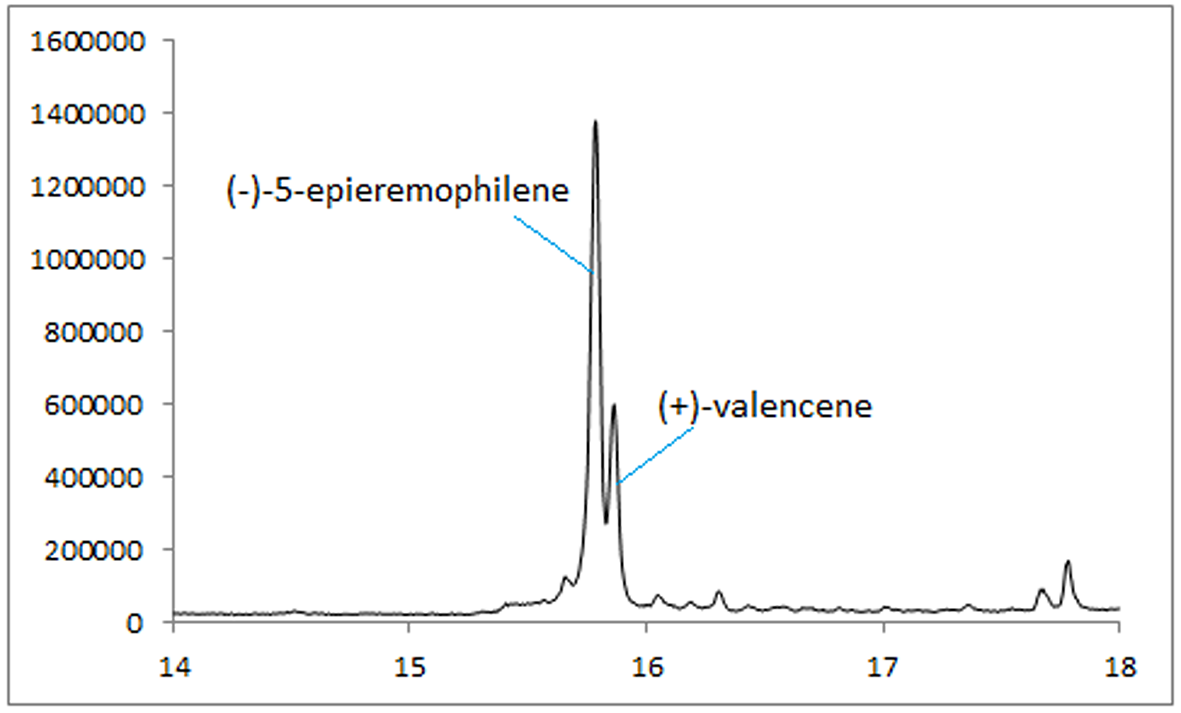

Supplement: FIGURE S2 — Total ion chromatograms from GC-MS analysis of the enzymatic product and authentic (+)-valencene. [file Image_2.TIF]

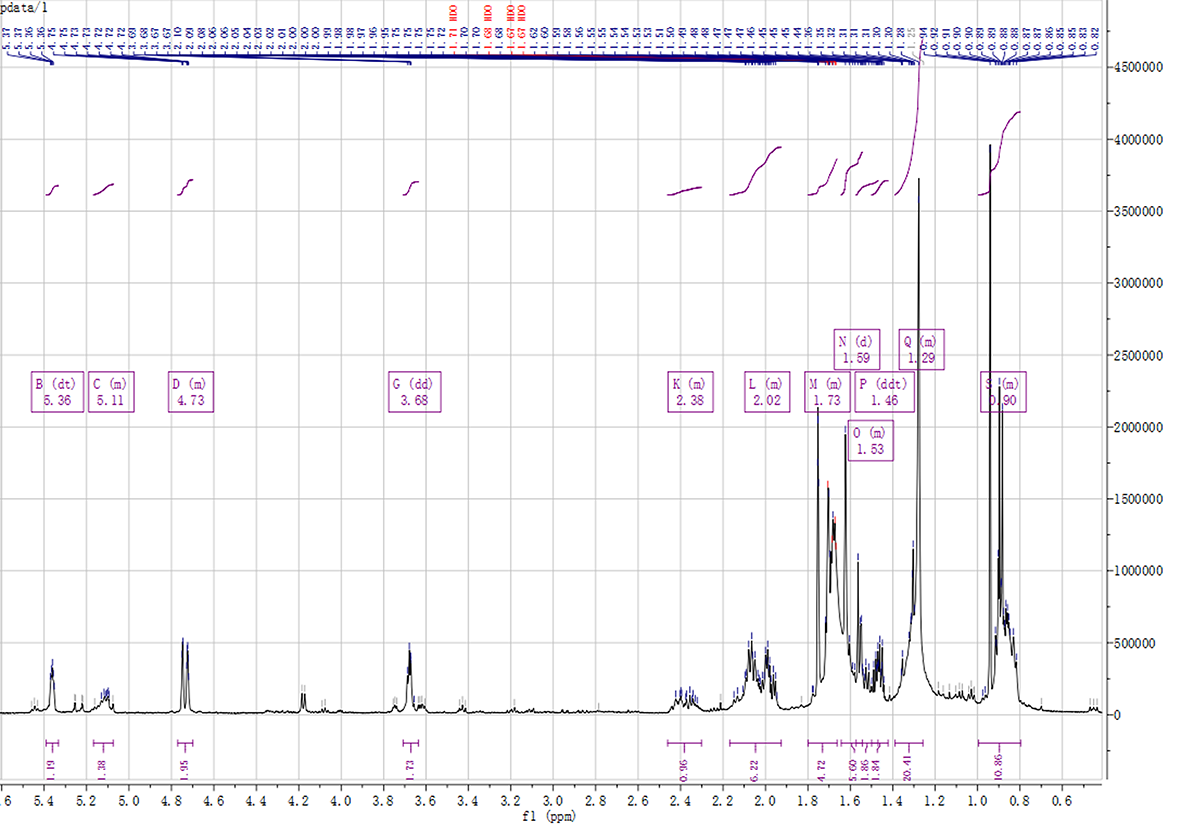

Supplement: FIGURE S3 — 1H NMR spectra of (-)-5-epieremophilene. [file Image_3.TIFF]

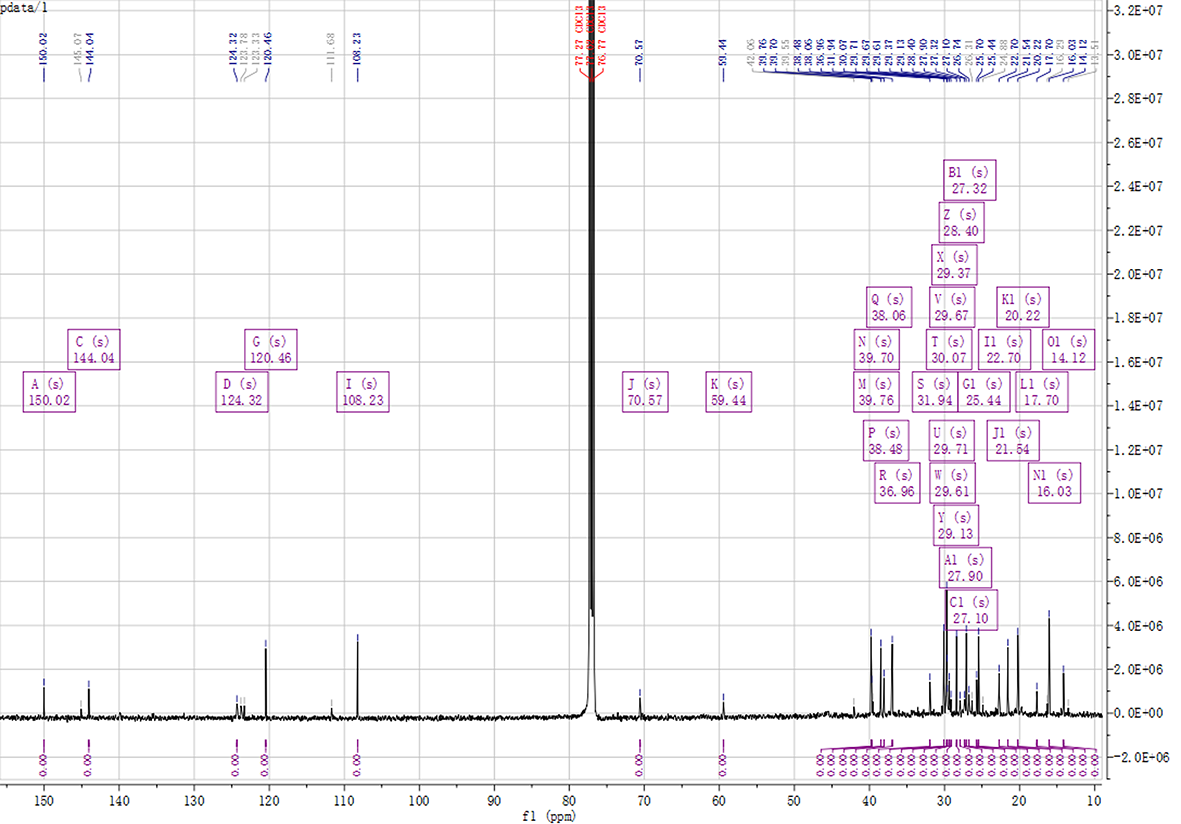

Supplement: FIGURE S4 — 13C NMR spectra of (-)-5-epieremophilene. [file Image_4.TIFF]

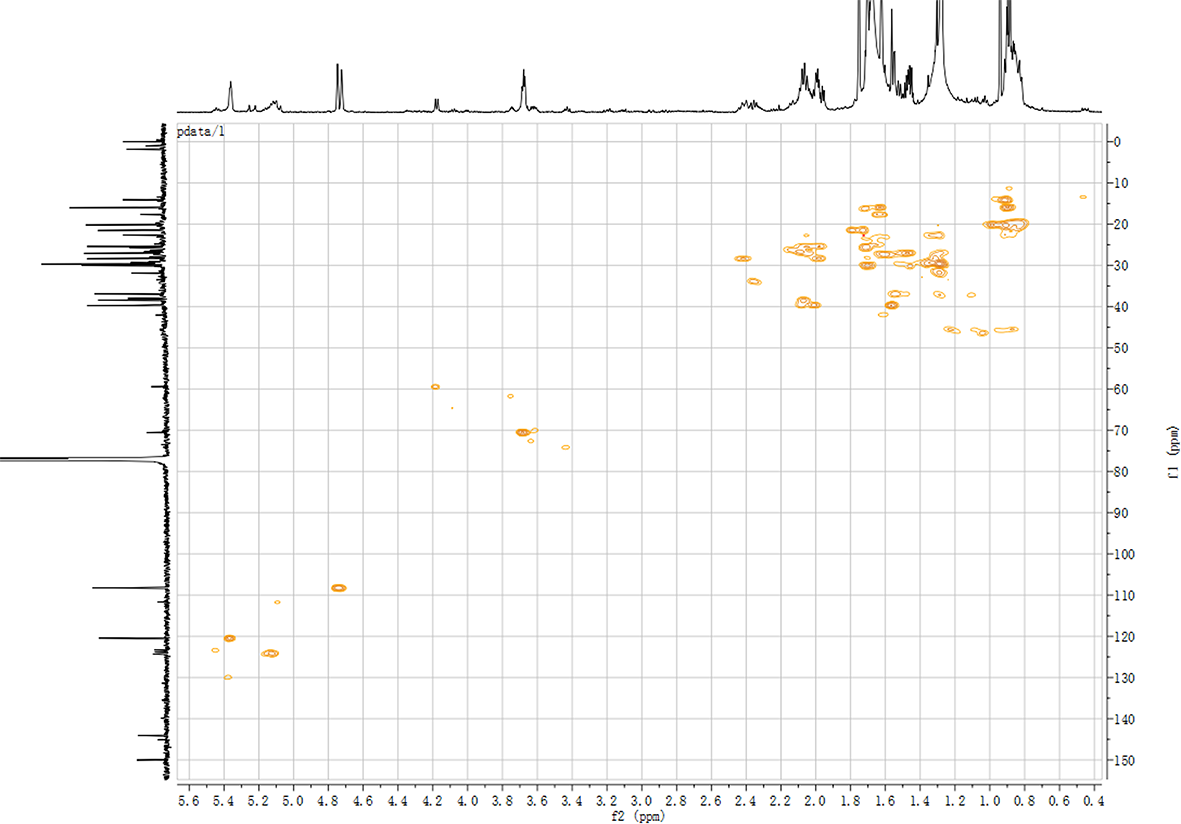

Supplement: FIGURE S5 — HSQC NMR spectra of (-)-5-epieremophilene. [file Image_5.TIFF]

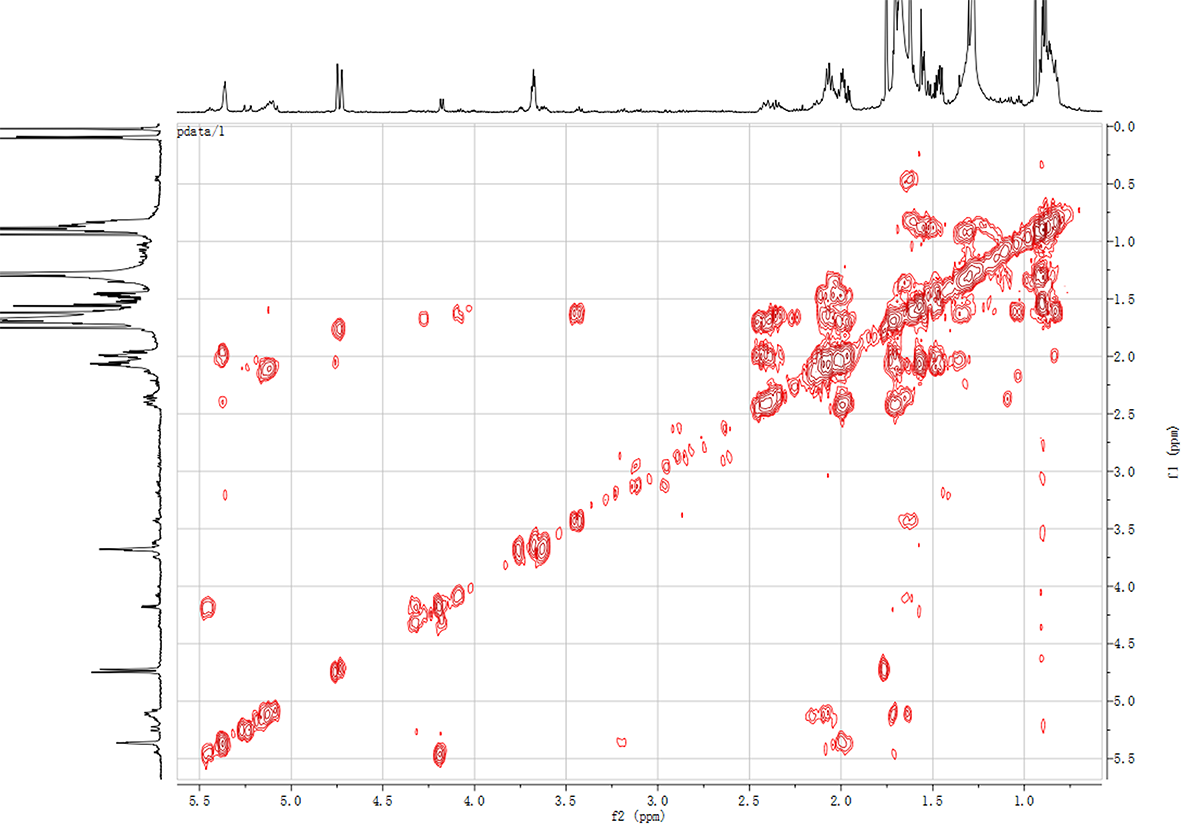

Supplement: FIGURE S6 — 1H-1H COZY NMR spectra of (-)-5-epieremophilene. [file Image_6.TIFF]

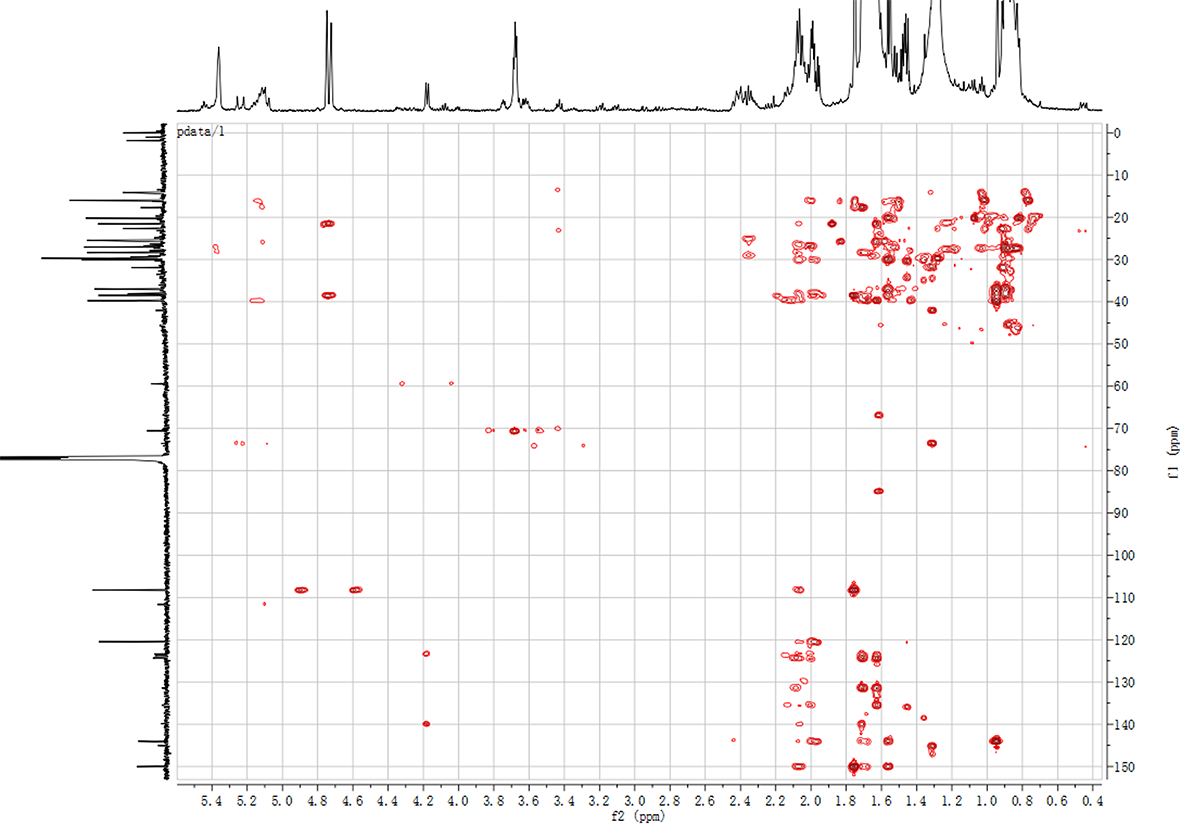

Supplement: FIGURE S7 — Heteronuclear multiple bond correlation HMBC NMR spectra of (-)-5-epieremophilene. [file Image_7.TIFF]

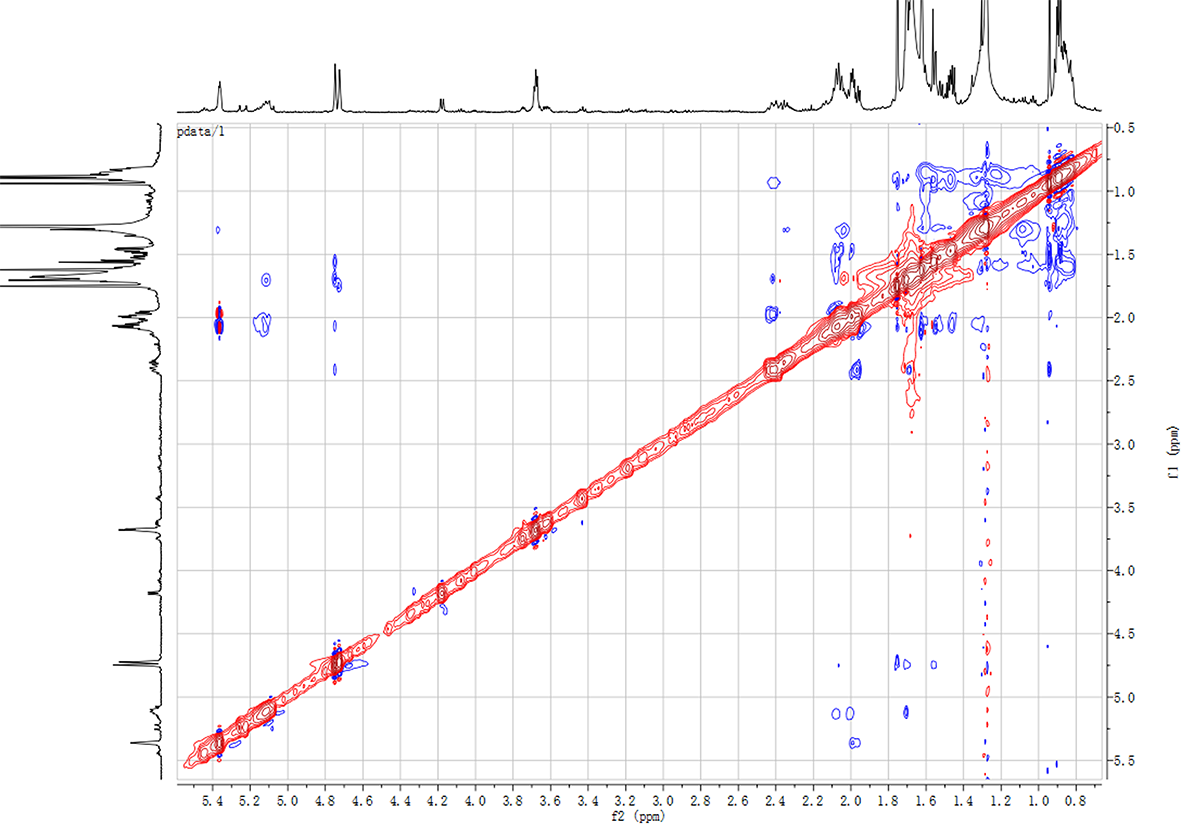

Supplement: FIGURE S8 — Rotating frame overhauser effect spectroscopy NMR spectra of (-)-5-epieremophilene. [file Image_8.TIFF]
